# The Effects of Pregnancy Status on Lacrimal Caruncle Temperature, Intraocular Pressure and Rectal Temperature in Cats: A Preliminary Study

**DOI:** 10.1002/vms3.70077

**Published:** 2024-10-17

**Authors:** Candemir Ozcan, Tarik Safak, Ayse Basak Dellalbasi, Elif Dogan

**Affiliations:** ^1^ Faculty of Veterinary Medicine, Department of Surgery Kastamonu University Kastamonu Türkiye; ^2^ Faculty of Veterinary Medicine, Department of Obstetrics and Gynecology Kastamonu University Kastamonu Türkiye

**Keywords:** feline, intraocular pressure, lacrimal caruncle temperature, pregnancy, thermal camera

## Abstract

**Objective:**

The objectives of this study were to compare the body temperatures between pregnant and nonpregnant cats from two sites, lacrimal caruncle temperature (LCT) and rectal temperature (RT), and to compare intraocular pressure (IOP) between pregnant and nonpregnant cats.

**Animal studied:**

This study was performed on 13 pregnant and 16 anoestrous cats.

**Procedures:**

The gestation period of the pregnant cats ranged from 20 to 45 days. A vaginal smear was also performed to determine the sexual cycles of nonpregnant cats. The IOP was measured using a rebound tonometer.

**Results:**

The pregnant cats (38 ± 0.7°C) exhibited a lower RT than the nonpregnant cats (38.5 ± 0.5°C) (*p* < 0.05). No significant differences existed between the pregnant and nonpregnant groups in the right (R)‐LCT or left (L)‐LCT (*p* > 0.05). The average LCT temperature measured 32.30 ± 2.23°C in cats. The right (R)‐IOP in pregnant cats (17.69 ± 5.6 mm Hg) was significantly lower than in nonpregnant cats (22.37 ± 5.27 mm Hg) (*p* < 0.029). Pregnant cats exhibited a significantly lower left (L)‐IOP value (17.69 ± 5.76 mm Hg) compared to nonpregnant cats (23.18 ± 5.55 mm Hg) (*p* < 0.015).

**Conclusion:**

This study presents a preliminary report that documents a noteworthy reduction in RT in pregnant cats (38 ± 0.7°C) as compared to cats in anoestrus. Pregnancy also has an effect on the IOP. It was hypothesised that the hormonal changes induced by pregnancy in cats would have a substantial impact on IOP and RT. Although body temperature can be measured using LCT, RT should still be utilised as the reference measurement site.

## Graphical abstract

• No significant differences existed between the pregnant and nonpregnant groups in right or left caruncle temperature.

• Right intraocular pressure (17.69 ± 5.6 mm Hg vs. 22.37 ± 5.27 mm Hg) and left intraocular pressure (17.69 ± 5.76 mm Hg vs. 23.18 ± 5.55 mm Hg) were both significantly lower in pregnant cats than in nonpregnant cats.

• Pregnant cats exhibited lower rectal temperature (38 ± 0.7°C) compared to nonpregnant cats (38.5 ± 0.5°C).

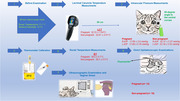

## 1 Introduction

During pregnancy, body temperature decreases shortly before parturition in different species such as rats, rabbits and sheep (Fewell 1995; Naccarato and Hunter 1983). The drop in body temperature towards the end of pregnancy is attributed to the readjustment of hypothalamic thermoregulatory neurones (Georgescu 2023). Furthermore, thermoregulatory patterns of core body temperature during pregnancy decrease with increasing duration of the gestation (Fewell 1995).

Various factors influence skin temperature patterns, with blood circulation being the most crucial. Dilation of skin capillaries, resulting from hyperaemia or inflammation, is associated with increased temperature, whereas constriction or loss of blood supply leads to a decrease in temperature (Sagaidachnyi et al. 2017). Infrared thermography is particularly effective in detecting subtle temperature variations on the face, especially in areas like the medial lower eyelid and the lacrimal caruncle, which respond autonomically to changes in blood flow (Kolosovas‐Machuca et al., 2016). The lacrimal caruncle, in particular, is frequently studied as a thermal window in animals (Stewart et al. 2008; Polat and Yanmaz 2024).

Intraocular pressure (IOP) is the outcome of the homeostasis between the generation and drainage of aqueous humour in the eye. The central nervous system effectively regulates and controls the IOP by maintaining an optimal balance between the production and outflow of aqueous humour (Okur et al. 2023). Factors such as extraocular muscle tone, eyelid manipulation, retractor bulbi muscle retraction, head/body position, external pressure, corneal curvature and thickness, corneal and scleral rigidity, intraocular changes, drugs, time of day and tear film viscosity can influence the assessment of IOP (Ofri and Horowitz 1998; Stodtmeister 1998; Shah 2000; Klein et al. 2011).

Acute glaucoma is classified as a critical eye condition that can result in irreversible vision loss if the IOP is not managed immediately. Tonometry is a useful method for measuring IOP, crucial for diagnosing severe eye conditions, including glaucoma and uveitis (Okur et al. 2023). IOP measurements were made by applanation tonometry in cats of different reproductive statuses. IOP in female cats in oestrus was significantly higher than IOP in female cats not in oestrus (Ofri et al. 2002). It is stated that pregnancy can reduce IOP in women with ocular hypertension and bring it to a normal range. It is thought that IOP decrease in ocular hypertensive pregnant women is related to high ocular pressure levels (Qureshi 1997). Progesterone concentrations are also reported to significantly reduce IOP in pregnant cats (Ofri et al. 2002).

The objectives of this study were to compare the body temperatures of pregnant and nonpregnant cats from two sites, lacrimal caruncle temperature (LCT) and rectal temperature (RT), and to compare IOP between pregnant and nonpregnant cats.

## 2 Materials and Methods

### 2.1 Animals

The material for the study consisted of cats brought to Kastamonu University, Faculty of Veterinary Medicine, Department of Obstetrics and Gynaecology for examination. The study comprised 29 female cats, 2 Scottish cats, 2 British short‐haired cats and 25 crossbred cats. Out of the total number of cats, 13 were pregnant, whereas 16 were nonpregnant (anoestrus). The ages were between 1.91 ± 1.37 (range: 1–7) years, and the body weights were between 3.15 ± 0.59 (range: 2.2–4.4) kg. Patients were evaluated in the examination room with their owners.

The female cats with the proper medical history, physical examination and disposition for the study were relocated to the designated area to measure body temperature (°C). Thus, it was aimed to make accurate digital and thermal measurements by minimising stress variables that may affect the results. Female cats with chronic conditions, gastrointestinal problems, acute infections or severe stress caused by their environment were excluded from this study.

### 2.2 Exclusion Criteria

Cats with chronic conditions, gastrointestinal problems, acute infections or severe stress caused by their environment were excluded from this study. Additionally, the cats underwent a thorough clinical examination by an animal surgeon to check for conditions like epiphora, lacrimation, eye discharge, blepharospasm, entropion and corneal erosion, which can potentially affect the corneal surface's temperature. This study did not include two pregnant cats with blepharospasm, one anoestrous cat with diarrhoea and one with ocular erosion.

The physicians who perform the pregnancy examination, the ocular examination and the temperature measurements are three distinct specialists. These specialists were unaware of the measurement outcomes to mitigate bias.

### 2.3 Environmental Conditions of the Examination Room

The female cats were confined in a designated part of the room for around 20 min to allow them to adjust before the measurements were taken (Slettedal and Ringvold 2015). There were only two proficient veterinarians in this specific area, specialising in the care of cats. All potential factors that could induce airflow in the space were effectively avoided. Temperature measurements were conducted in a room with a consistent temperature of 25.5°C (minimum 25, maximum 26) and a relative humidity (RH) of 58.0% (minimum 51, maximum 65). The room was not exposed to direct sunlight. Isolated room temperature and RH were recorded using the device positioned at an elevated level of ∼ 3 m above the ground. LCT was assessed using a thermal camera. RT was analysed and assessed using a digital thermometer. All measurements were simultaneously recorded. The time from 09:00 AM to 11:00 AM is a range within the day.

### 2.4 Monitoring

A patient monitor (Comen C80‐V, Shenzhen, China) was used to measure pulse and respiratory rate. As previously reported by Şenocak (2023), electrocardiogram leads were connected to the front and left hind legs, and the data were recorded.

### 2.5 Measurement of Lacrimal Caruncle Temperature

**FIGURE 1:**
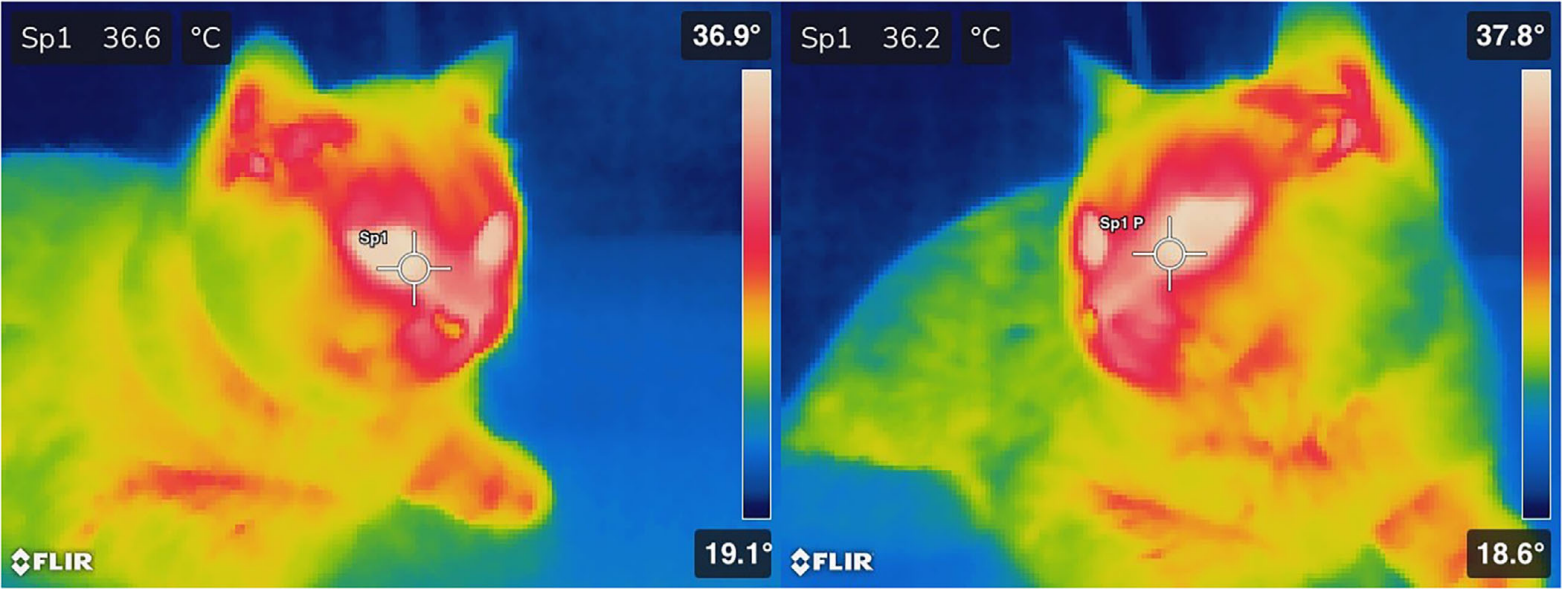
Measurement of lacrimal caruncle temperature in a cat with the thermal camera.

After acclimating the animals to the environment, temperature measurement with a thermal camera (FLIR E90 160 × 120 pixel, Systems, Inc., Sweden) was preferred as the first measurement, on the grounds that RT measurement or eye pressure measurement could create stress in the animal and affect the results of the thermal camera measurement. The most commonly studied thermal window in animals is the lacrimal cartilage (Stewart et al. 2008). For this reason, our location of choice for thermal camera measurement is the lacrimal cartilage. Measurements were made from the lacrimal cartilage symmetrically, from equal distances, with a thermal camera (Figure 1). The same person always took lacrimal cartilage thermograms without touching the animals and moving slowly to prevent stress. The thermal images were taken using a thermal camera positioned at a distance of 90 cm. Two people were present in the examination room, including the veterinarian who took the images.

### 2.6 Intraocular Pressure Measurement

**FIGURE 2:**
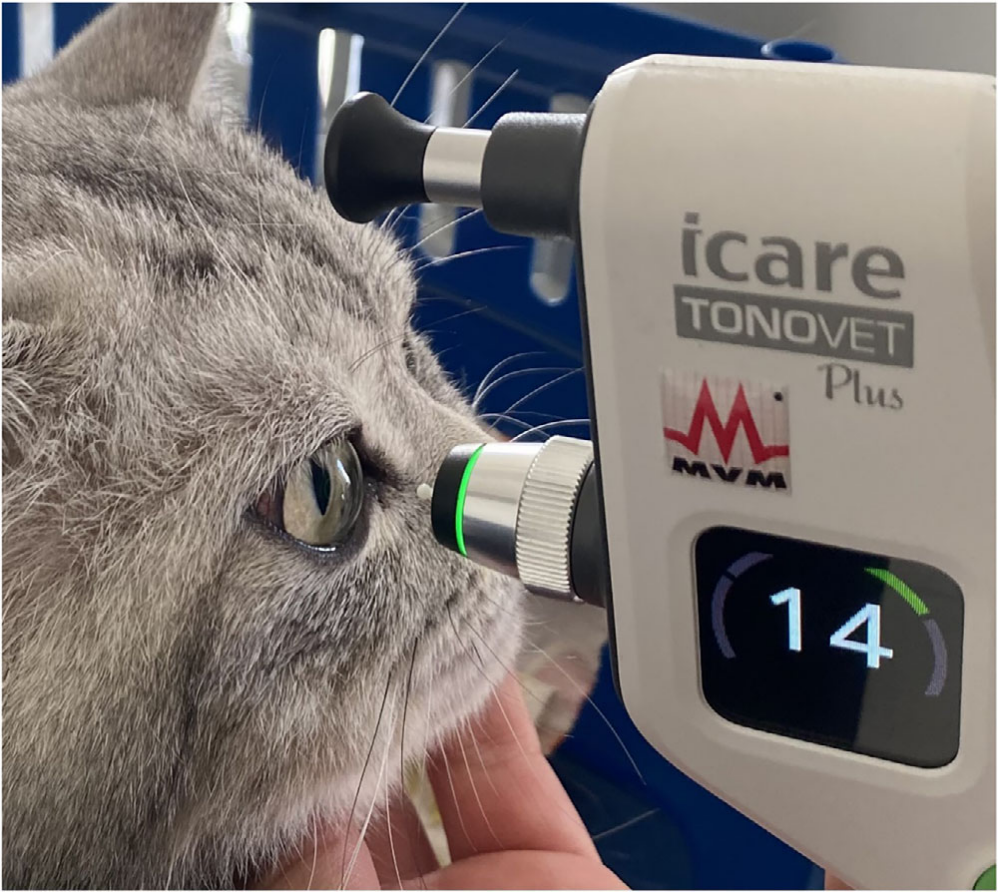
Measurement of intraocular pressure in a cat with a tonometer.

The IOP was measured using a rebound tonometer (TonoVet Plus, Jorgensen Laboratories, Loveland, CO, USA) (Figure 2). Tonometry has the ability to calibrate itself before each use. The IOP measurements were conducted by one examiner, positioned at a 90° angle to the central cornea. IOP was initially measured from the right eye in each cat. The tonometer recorded six readings in succession and then displayed the average IOP in mm Hg. The measurements were repeated until the tonometer showed that a satisfactory level of consistency was achieved among the six measurements, as indicated by an acceptable standard deviation (± 5%) (Rajaei 2018).

### 2.7 Measurement of Rectal Temperature

The study conducted by Giannetto et al. (2021) was used as a reference value for the measurement of RT. A previously calibrated digital thermometer (Albert KERBL, GmbH, Germany) was used for each measurement. The probe was placed about 3 cm into the rectum. The device's measurement range extends from 32.0°C to 42.0°C, with a precision of ± 0.1°C. This rectal thermometer has a rapid response time of ∼ 9–11 s and produces an audible alert once the maximum temperature is reached. Upon detecting the signal from the thermometer, it was removed from the rectum, and the data were subsequently recorded on the display. Glycerine was applied to the probe of the digital thermometer before insertion into the rectum.

### 2.8 Ocular Examination

To assess the presence of erosion on the ocular surface of the female cats, an exam known as fluorescein sodium staining (Fluorescite Ophthalmic Disclosing Agent Fluorescein Sodium 10%) was conducted by Kaya et al. (2023).

### 2.9 Ultrasonographic Evaluation of the Genital Organs and Vaginal Smears

The ultrasound evaluation followed the protocols described by Pecchia et al. (2023). A 5 MHz convex probe in B‐mode (Versana Active, USA) was used for the transabdominal ultrasound test to determine if the cats were pregnant. The gestation period of the pregnant cats ranged from 20 to 45 days. The ovaries and uterus were visualised during the ventro‐dorsal position. Cats with maternal‐foetal structures were included in the pregnant group (Safak and Yilmaz, 2023). A vaginal smear was also performed to determine the sexual cycles of nonpregnant cats.

Vaginal smears and the determination of sexual cycles were performed as described by Termelioğlu, Kalender, and Erat (2022). For the vaginal smear, cells taken from the vaginal tissue with a cotton swab were spread on a clean and pre‐labelled slide. The cells collected from the vaginal area were air‐dried and fixed with ethyl alcohol added to the slide. Then, the slides were covered entirely with Giemsa stain using a dropper so that it was evenly distributed in each region. After the vaginal smear preparations stained with Giemsa were allowed to dry in the air, they were examined under a light microscope (Leica DM500, Germany). The cells were categorised based on their morphology, which is parabasal, intermediate, superficial or keratinised superficial. Cats in an anoestrus period due to cell profiles were used as a nonpregnant group.

### 2.10 Statistical Analysis

The statistical analyses were performed using the IBM SPSS 22.0 software. The data received a normality assessment using the Shapiro–Wilk test. The independent samples *t*‐test was used because the weight, RT, respiration frequency, right intraocular pressure (R‐IOP), left intraocular pressure (L‐IOP), R‐LCT and L‐LCT were all normally distributed. Body temperatures were obtained through various methods, such as measuring the LCT with the rectal measurement and reference method, to conduct Bland–Altman plot analysis (Bland and Altman 1986). Linear regression analyses were conducted utilising the disparities and the average outcomes of the measurements. The significance was determined when the *p*‐value was < 0.05. The correlation was determined using the Pearson or Spearman correlation test, depending on the data distribution. If the data has a parametric distribution, the Pearson correlation test was employed; otherwise, the Spearman correlation test was utilised. The analysis results were considered statistically significant if the *p*‐value was < 0.05. The results were reported as the mean ± standard deviation. Cohen's *d* values were calculated to determine the effect size of the variables (Cohen 1988, 531–542).

## 3 Results

The RT levels exhibited a statistically significant difference between the groups. The pregnant cats (38 ± 0.7°C) exhibited a lower RT than the nonpregnant cats (38.5 ± 0.5°C) (*p* = 0.034). The effect size (Cohen's *d*) for RT was calculated as 0.76 (95% CI, 0.00–1.52). The linear regression analysis revealed a positive correlation between RT and body weight (*p* = 0.027, *R* ^2^ = 0.169). No significant differences were observed between the groups in terms of age, body weight, respiratory frequency, LCT, and pulse rate (p > 0.05) (Table 1).

**TABLE 1:** Grouped data on age, body weight, RT, LCT, respiration frequency and pulse rate.

Groups	*N*	Age (year)	Body weight (kg)	RT (°C)	LCT (°C)	Respiration (/min)	Pulsation (frequency/min)
**Pregnant**	13	2.4 ± 1.7	3.27 ± 0.62	38.0 ± 0.7	32.55 ± 1.64	47 ± 16	148 ± 45
**Nonpregnant**	16	1.5 ± 0.7	3.06 ± 0.56	38.5 ± 0.5	32.81 ± 2.01	52 ± 11	140 ± 32
***p*‐value**		—	—	*	—	—	—

Abbreviations: LCT, lacrimal caruncle temperature; *n*, number of cases; RT, rectal temperature.

–: *p* > 0.05; *p < 0.05.

The pulse rate of pregnant cats was 148 ± 45.37 beats per minute, and that of nonpregnant cats was 140.62 ± 32.10. Pulse rates between groups were not statistically significant (*p* > 0.05).

**FIGURE 3:**
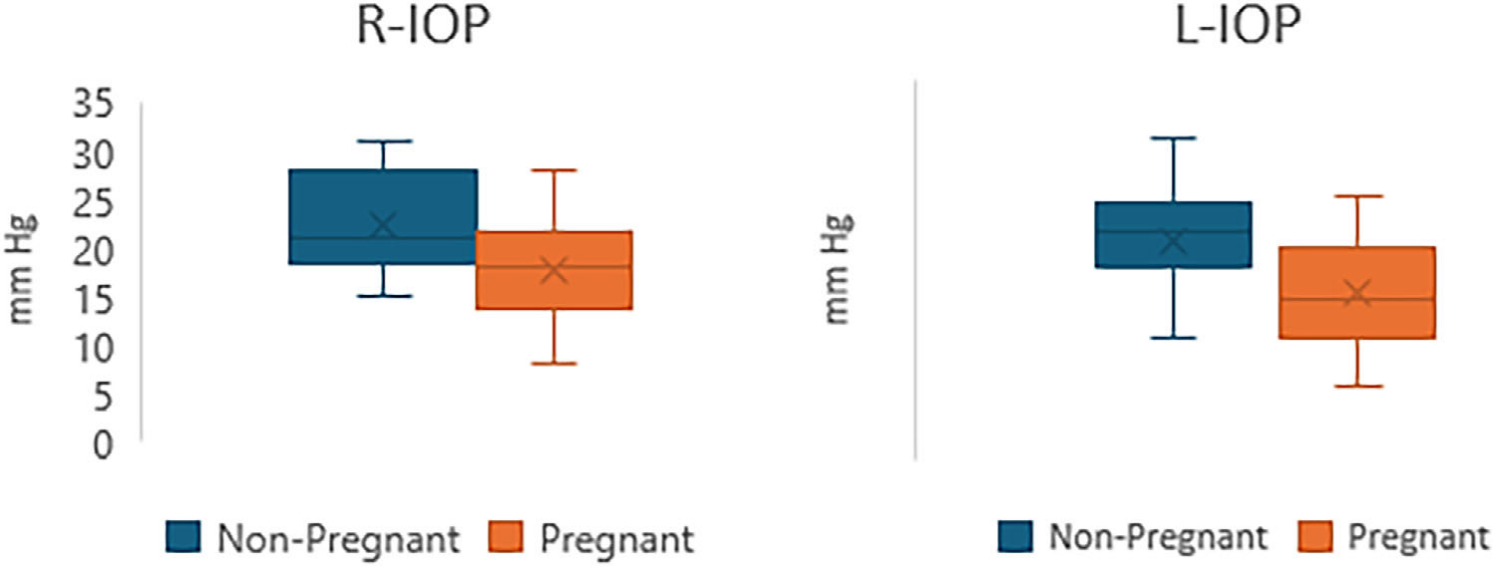
Mean and range values of R‐IOP and L‐IOP in cats categorised by groups.

The R‐IOP in pregnant cats (17.69 ± 5.6 mm Hg) was significantly lower than in nonpregnant cats (22.37 ± 5.27 mm Hg) (*p* = 0.029) (Cohen's *d* = 1.14, 95% CI, 0.35–1.93). Pregnant cats exhibited a significantly lower L‐IOP value (17.69 ± 5.76 mm Hg) compared to nonpregnant cats (23.18 ± 5.55 mm Hg) (*p* = 0.015) (Cohen's *d* = 1.51, 95% CI, 0.68–2.34). The R‐IOP and L‐IOP levels for each group are presented in Table 2. The mean and range values of R‐IOP and L‐IOP in cats, categorised by groups, are shown in Figure 3.

**TABLE 2:** Levels of R‐IOP and L‐IOP categorised by groups.

Groups	*n*	R‐IOP	L‐IOP	*p*‐value
**Pregnant**	13	17.69 ± 5.6	17.69 ± 5.76	—
**Nonpregnant**	16	22.37 ± 5.27	23.18 ± 5.55	—
***p*‐value**		*	*	

Abbreviations: L‐IOP, left intraocular pressure; *n*, number of cases; R‐IOP, right intraocular pressure.

–: *p* > 0.05; *p < 0.05.

**FIGURE 4:**
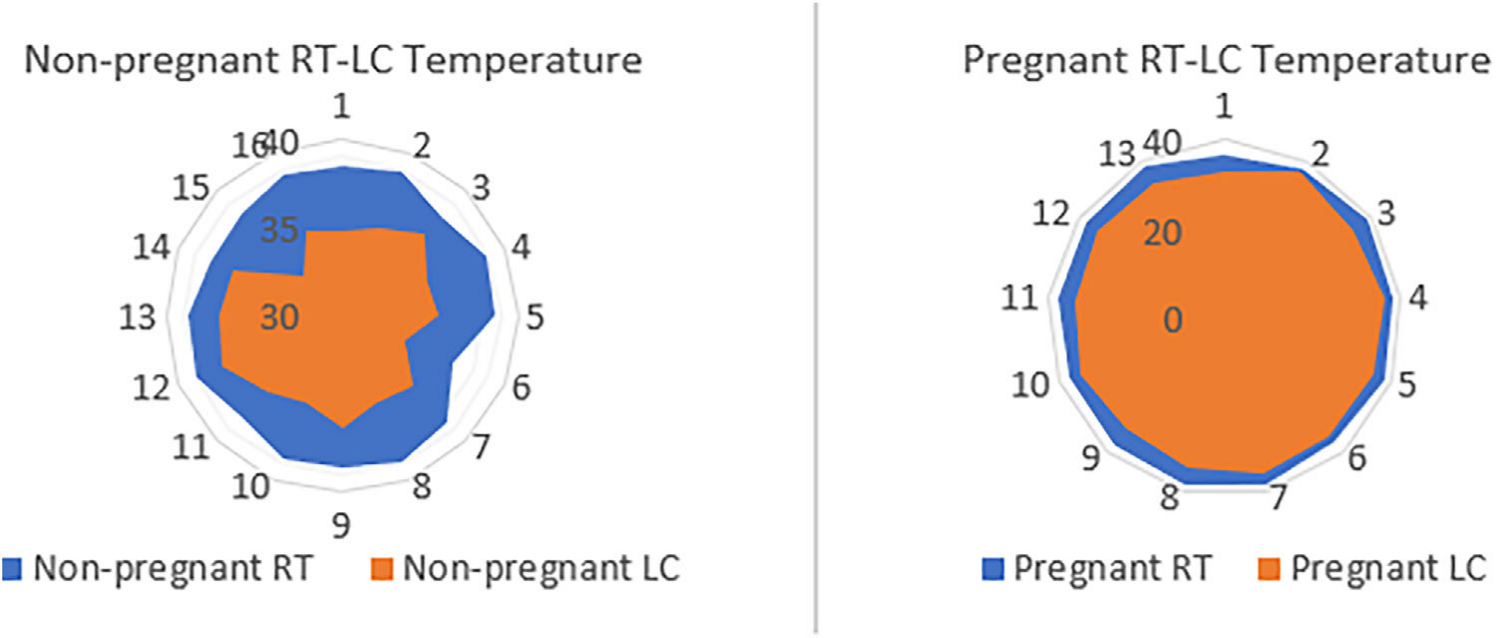
Graph showing the differences in temperature between the RT (blue zone) and the LCT (red zone) of pregnant and nonpregnant cats.

In pregnant cats, R‐LCT is 32.60 ± 2.10°C and LCT is 32.51 ± 1.67°C (Cohen's *d* = 0.16, 95% CI, −0.57 to 0.89). In nonpregnant cats, R‐LCT is 32.85 ± 2.23°C and LCT is 32.78 ± 2°C (Cohen's *d* = 0.04, 95% CI, −0.69 to 0.77). No significant differences existed between the pregnant and nonpregnant groups in the R‐LCT or L‐LCT (*p* > 0.05). However, there exists a substantial (*p* < 0.0001) and positive correlation (*r* = 0.704) between R‐LCT and L‐LCT temperatures. Therefore, the R‐LCT and L‐LCT temperatures of each animal were averaged. The average LCT temperature measured 32.30 ± 2.23°C in cats. The mean RT of cats was 38.23 ± 0.65°C. There was a statistically significant difference between LCT and RT (*p* < 0.001). The temperature distribution of these average LCT levels according to RT is shown in Figure 4. The study found no statistically significant correlation between IOP levels and LCT temperatures (*p* > 0.05).

The regression analysis results show that the model has partially explains the relationship between RT and IOP, but since the p‐value obtained is > 0.05, it is concluded that this relationship is not statistically significant and the effect of RT on IOP is most likely coincidental (*p* > 0.05, *R* ^2^ = 0.26).

**FIGURE 5:**
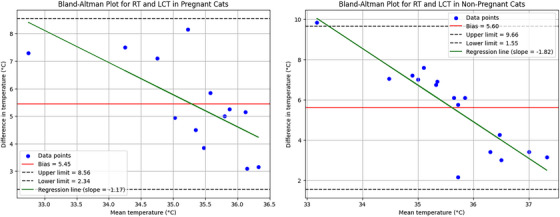
Bland–Altman plot analysis graph between RT‐Mean LCT values of pregnant (left graph) and nonpregnant (right graph) cats. In the graph, the red line represents bias, the green line represents the regression slope, the black dashed line represents the upper and lower limits and the blue dots represent the data points.

The Bland–Altman analysis was conducted to assess the agreement between RT and LCT in pregnant and nonpregnant cats. In pregnant cats, the analysis revealed a bias of 5.45, with upper and lower limits of agreement calculated as 8.56 and 2.34, respectively. The regression line slope was determined to be −1.17. Similarly, for nonpregnant cats, the bias was 5.60, with upper and lower limits of 9.66 and 1.55, respectively. The regression slope for nonpregnant cats was more pronounced at −1.82, suggesting a stronger negative relationship between the differences and mean temperatures. (Figure 5).

## 4 Discussion

This research indicates that the RT of pregnant cats is lower than that of nonpregnant cats. The observed temperature difference exhibited statistical significance. The results demonstrate that pregnancy can affect the body temperature of cats, as evidenced by the lower RT reported in pregnant cats compared to nonpregnant cats. As in previous studies conducted in humans (Hartgill, Bergersen, and Pirhonen 2011) and animals (Gamo et al. 2013), a decrease in body temperature during pregnancy is parallel to the findings of this study. During the first stage of pregnancy in killer whales, there is a simultaneous rise in body temperature and progesterone levels. Nevertheless, there is a progressive decrease in temperature leading up to the end of parturition, with the most significant decrease occurring on the day before parturition (0.8°C) (Katsumata et al. 2006).

The decrease in body temperature could also be attributed to hormonal changes that occur during pregnancy. Estradiol‐17β, luteinising hormone and progesterone are the primary hormones released at different rates during pregnancy in female cats (Kustritz 2006). The relationship between body temperature and indicators such as oestrus, pregnancy and parturition has been extensively studied in several domesticated animals, including cattle, sheep, horses and dogs (Ewbank 1969; Godyń, Herbut, and Angrecka 2019; Verstegen‐Onclin and Verstegen 2008; Auclair‐Ronzaud et al. 2020; Kaya et al. 2023). Changes in body temperature in cattle during pregnancy are linked to variations in hormone levels, particularly progesterone levels, because of the thermogenic impact of progesterone (Kornmatitsuk et al. 2002; Nabenishi and Yamazaki 2017; Suthar et al. 2012). The corpus luteum and/or placenta are the main sources of the steroid hormone progesterone, which significantly impacts the oestrous cycle and the start and maintenance of pregnancy (Wiltbank et al. 2014). Throughout pregnancy, the corpus luteum remains intact and secretes elevated quantities of progesterone, leading to a rise in vaginal temperature (Suthar et al. 2012; Wiltbank et al. 2014). This shows that different clinical findings may be encountered in different animal species. It also reveals that different body temperature patterns are observed in different anatomical regions.

It is important to acknowledge that this study focused primarily on female cats and may not include all mammal species. It is also reported that different results can be obtained when different animal species live in different environmental conditions. It is essential to consider other factors affecting body temperature, such as the environment and activity levels. Pregnant moose (*Alces alces*) have a body temperature that is 0.13°C–0.19°C higher than nonpregnant moose, and the body temperature decreases as parturition nears (Græsli et al. 2022). Assuming all other factors remain constant, an arctic animal living at a temperature of −10°C exhibits a body temperature that is around 2.7°C higher and a basal metabolic rate that is almost 40% more than that of a tropical mammal of the same size residing in a temperature of 25°C (Clarke, Rothery, and Isaac 2010). It is possible that pregnant cats may exhibit a lower body temperature due to reduced physical activity and increased time spent resting. Additionally, individual differences and genetic factors within each species could have an impact on variations in body temperature during pregnancy (Zambelli 2014).

This study's results show a negative correlation between RT and body weight in cats. As the body weight increases, one might expect the body temperature to rise; however, the study's findings suggest that pregnancy's higher metabolic needs and hormonal changes are to blame for the observed temperature drop (Kustritz 2006; Holst 2022). It is crucial to consider the possible influence of hormones on regulating body temperature during pregnancy (Ofri et al. 1999). Evidence suggests that blood progesterone levels may not directly regulate body temperature but rather a combination of oestrogen and progesterone may have a synergistic role (Forman et al., 1987). Progesterone has a calming effect on the central nervous system, probably leading to a reduction in body temperature. In addition, higher levels of hormones such as oestrogen and oxytocin, which are also present during pregnancy, may influence the regulation of body temperature (Schmidt, Chakraborty, and Wildt 1983).

In this study, both right and left eye IOP were lower in pregnant cats than in nonpregnant cats. Systolic and diastolic blood pressures, body weight, height or the number of previous pregnancies in women do not affect reductions in IOP. Female cats in oestrus had significantly higher IOP compared to female cats not in oestrus. Progesterone levels had a significant impact on IOP in pregnant cats (Ofri et al. 2002). The greater reduction in ocular hypertensive individuals may be attributed to their elevated ocular pressure levels. Pregnancy can lower IOP in individuals with ocular hypertension, bringing it within the normal range (Qureshi 1997). Epidemiological studies indicate a correlation between IOP and systemic blood pressure. However, according to Ebeigbe, Ebeigbe, and Ighoroje (2012), this is not attributed to systemic blood pressure. Instead, they found a positive link between IOP and both systolic and diastolic blood pressure during pregnancy. It is critical to understand how variations in heart rate and blood pressure affect IOP. It must be identified whether this is related to changes in heart rate/blood pressure or tissue remodelling induced by disorders like glaucoma (Jin et al. 2020). Our data demonstrated no relationship between IOP and pulse rate in female cats, highlighting the complexities of the connections between circulatory dynamics and ocular health.

Since there was no evidence of any statistically significant differences among symmetrical organs (R‐LCT and L‐LCT) in this investigation, temperature averages were considered. This result is in line with the findings of the research conducted on cats (Giannetto et al. 2021). The average RT in this research of female cats was 38.23 ± 0.65°C, while the average LCT was 32.30 ± 2.23°C. This suggests that there is a significant temperature differential between the two areas. However, in the present study, the LCT temperature was not sensitive enough to identify the difference between pregnant and nonpregnant cats, even though RT demonstrated a statistically significant difference between the two groups. The mean (± SD) RT and LCT temperature of female cats were 38.23 ± 0.65°C and 32.30 ± 2.23°C, respectively. There was a statistically significant difference between the temperatures of these different regions (*p* < 0.001). There is no doubt that this may be due to the difference in the measurement method. Variations in the environment's temperature have an even greater effect on the LCT temperature. This occurs due to the fact that the LCT is anatomically in contact with the air in the environment. A statistically significant temperature difference was found between LCT and mean RT in female cats. This is why the rectal area is more isolated and positioned within the body than the LCT.

However, it is important to note that there was no significant difference in LCT between the groups. The LCT is considered the most thermally active component of the ocular region (Stewart et al. 2008). The ocular regions, particularly the posterior margin of the eyelid and the area around the lacrimal caruncle, contain rich capillary networks that respond to changes in blood flow and cause localised temperature fluctuations in humans (Pavlidis, Eberhardt, and Levine 2002), cattle (Stewart et al. 2007) and dogs (Biondi et al. 2015). The mentioned literature above shows why the LCT in pregnant and nonpregnant cats has different thermoregulatory properties in terms of RT.

According to Giannetto et al. (2021), there is a connection between ocular temperature and RT. Nonetheless, the data in this study's Bland–Altman analysis graph show an irregular pattern. This shows that LCT temperature shouldn't be substituted for RT temperature in female cats. The bias value calculated according to pregnancy status was 5.45°C for pregnant cats and 5.60°C for nonpregnant cats. These findings indicate that there is a systematic difference between temperature measurements in both groups. When the upper limit (8.56°C) and lower limit (2.34°C) observed in pregnant cats were compared with the upper limit (9.66°C) and lower limit (1.55°C) in nonpregnant cats, it was observed that the measurement variance in nonpregnant cats had a wider range. This indicates a greater variability of measurements in nonpregnant cats. According to the regression slope results, the slope was −1.17 in pregnant cats and −1.82 in nonpregnant cats. These negative slope values indicate that the measurement differences decrease with the mean temperature and that the differences become smaller as the mean temperature increases. Although this trend indicates that temperature differences in both pregnant and nonpregnant cats generally follow a similar direction, this decrease is more pronounced in nonpregnant cats.

Regarding the LCT in cats, it has been reported that this region is sensitive in assessing stress levels (Casas‐Alvarado et al. 2022). In this study, LCT did not show a statistical difference between pregnant and nonpregnant cats. This finding implies that stress may not be the only factor contributing to the statistically significant difference observed in RT and IOP levels between pregnant and nonpregnant cats. The study found no statistically significant correlation between IOP levels and LCT temperatures. This finding represents a significant result of the investigation. One of the primary conclusions of the research is that there is no statistically significant link between IOP levels and LCT. Despite measuring IOP and LCT at several anatomical regions, no significant association was seen between the two variables. These findings are important because LCT does not impact IOP, which is crucial in clinical practice. Additionally, while the RT of pregnant cats decreased significantly compared to nonpregnant cats, the lack of a significant difference in the LCT data between the two groups suggests that either the LCT measurement method is less reliable than the RT method or that the sample size is insufficient. Specifically, conducting more comprehensive investigations to assess the impact of various factors or circumstances (such as stress levels and hormonal fluctuations) on IOP and LCT might be beneficial.

The completion of the comprehensive eye examination is an essential step that must be taken before beginning the process of classifying glaucoma and putting suitable treatment measures into place (Grahn 2023). It is important to assess factors contributing to elevated IOP, such as uveitis or lens luxation, to accurately diagnose and manage glaucoma in cats (Czederpiltz et al. 2005). Nevertheless, cats with different levels of IOP were identified. Therefore, it was aimed to minimise measurement differences due to anatomical variation. Cats with IOPs at the upper limit stated in the literature (Grahn 2023) should be carefully monitored after pregnancy.

This preliminary study has certain limitations that should be acknowledged. One of the primary limitations is the relatively small sample size, which was a result of the study being conducted on clinical cases that presented at our facility. The limited number of cases may reduce the generalisability of the findings and could impact the statistical power of the results. Consequently, the outcomes observed in this study may not be fully representative of the broader population. Future studies with larger and more diverse samples are necessary to confirm and expand upon these findings.

## 5 Conclusions

This preliminary study demonstrates that pregnancy significantly lowers RT in cats compared to nonpregnant ones. IOP was also found to be lower in pregnant cats, suggesting an impact of pregnancy on ocular physiology. However, no significant differences were observed in LCT between the groups. Importantly, monitoring body temperature in pregnant cats is critical, as elevated temperatures may pose health risks. Careful management of thermoregulation is recommended to prevent potentially dangerous hyperthermia in pregnant cats.

## Author Contributions

**Candemir Ozcan**: investigation, resources, writing manuscript draft, review and editing. **Tarık Safak**: Performed pregnancy examinations, determined sexual cycle periods, review and editing. **Ayse Basak Kapcak**: data collection. **Elif Dogan**: data collection, final checks, editing. All authors read and approved the final manuscript.

## Ethics Statement

The measurements and examinations conducted on the female cats adhered to the guidelines set by the Association for Research in Vision and Ophthalmology to utilise animals in ophthalmic and vision research properly. Cat owners approved the consent form to perform the treatment on the cats included in the study. This study was also conducted following approval by the Kastamonu University Local Ethics Committee of Animal Experimentation (date: 10.01.2024, approval number: 2024/01).

## Conflicts of Interest

The authors declare no conflicts of interest.

### Peer Review

The peer review history for this article is available at https://www.webofscience.com/api/gateway/wos/peer-review/10.1002/vms3.70077.

## Data Availability

The data that support the findings of this study are available from the corresponding author, upon reasonable request.
